# Supplementary material for: Overcoming Immunological Resistance Enhances the Efficacy of a Novel Anti-tMUC1-CAR T Cell Treatment against Pancreatic Ductal Adenocarcinoma
Source: Cells. 2019 Sep 11;8(9):1070. doi: 10.3390/cells8091070 (PMC6770201; doi:10.3390/cells8091070)
Supplement: Supplementary file 1 [file cells-08-01070-s001.zip › cells-588476 Supplementary Data Proof Back copy.pdf]

Supplementary Data

Supplementary data: video. Dynamic interaction of CAR-T cells and PDA target cells (BxPC3-MUC1 vs. BxPC3-Neo cells) recorded by time laps imaging using GE DeltaVision OMX-SR imaging system. Cancer cells and CAR T cells were co-cultured in 35 mm MatTek dish and placed in the microscope's 37 °C 5% CO2 incubator overnight. Images were taken from 11 spots over the course of 8 h at 7 min intervals. Red staining is indicative of dead cells that have absorbed propidium iodide dye present in the media. Videos illustrate that BxPC3-MUC1 cells were aggressively destroyed by CAR T cells, while BxPC3-Neo cells were mostly intact at 1:10 T:E ratio. Pictures were analyzed using ImageJ v1.51f program.

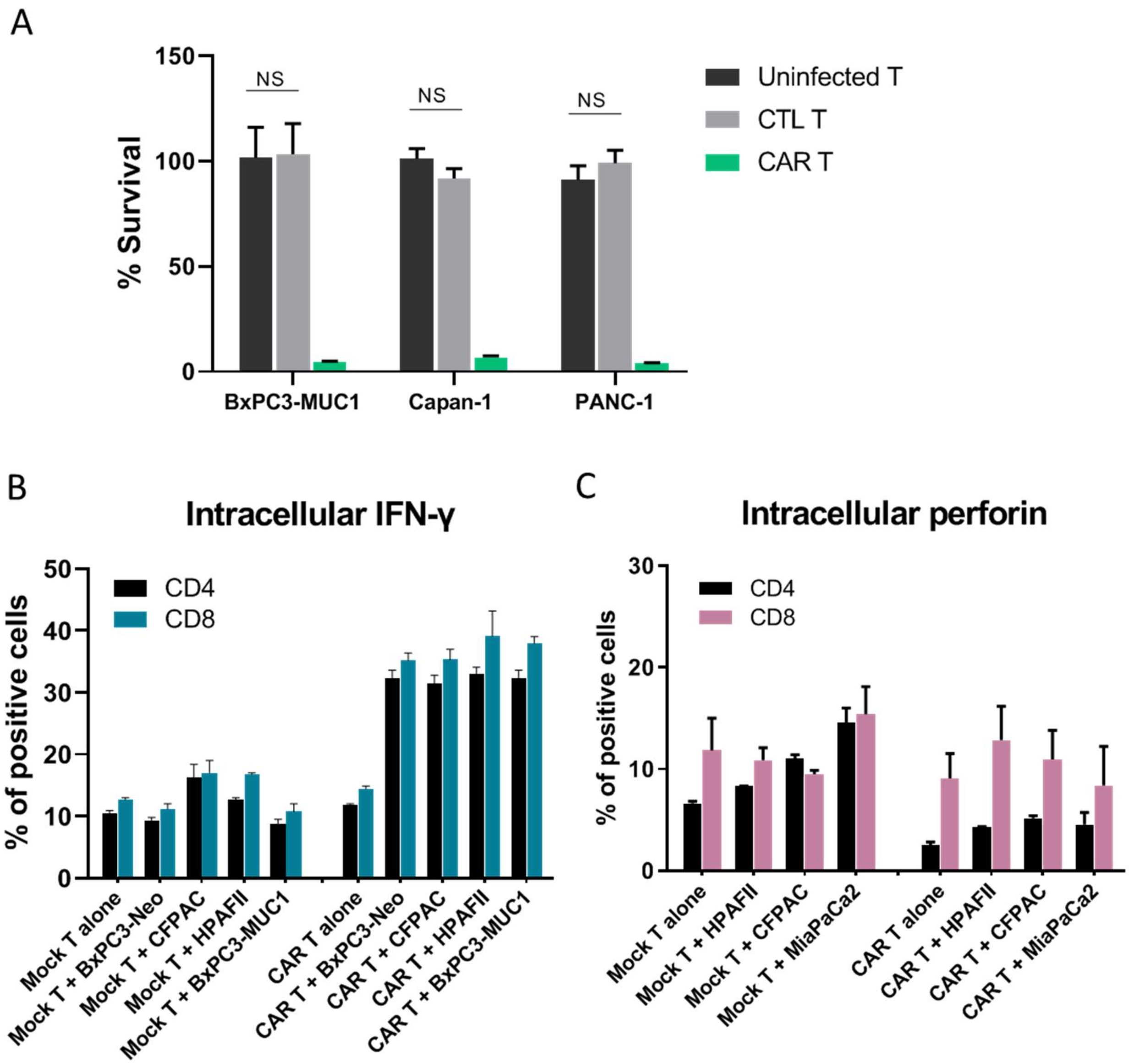

Figure S1. A. Similar activity of uninfected T and CTL T cells. Uninfected, CTL, or CAR T cells were exposed to indicated PDA cell lines for 72 h at T:E 1:10, and the survival level of PDA cells were measured using MTT assay. Data was normalized to media alone. Uninfected and CTL T show similar killing ability against PDA cells. Student's t-test, NS $P > 0.05$. **B, C.** Intracellular level of IFN-γ **(B)** and perforin **(C)** in mock and CAR T cells before and after exposure to PDA cells for 24 h at T:E 1:10. Percentage of $CD4^+$ and $CD8^+$ CAR T cells that are positive for IFN-γ and perforin was measured by flowcytometry. Data suggests CAR T cells exposed to HPAFII and CFPAC resistant cells are not functionally impaired regarding the production of IFN-γ and perforin internally. One-way ANOVA comparing the mean of each group showed no significant difference between the amount of intracellular IFN-γ produced by CAR T cells in BxPC3-Neo, CFPAC, HPAFII, and BxPC3-MUC1 groups (P value for CD4 = 0.8362, P value for CD8 = 0.4691). Similarly, one-way ANOVA did not detect any significant difference between the amount of intracellular perforin produced by CAR T cells in HPAFII, CFPAC, and MiaPaCa2 groups. P value for CD4 = 0.7102, P value for CD8 = 0.6761. Error bars, SEM. $n = 3$.

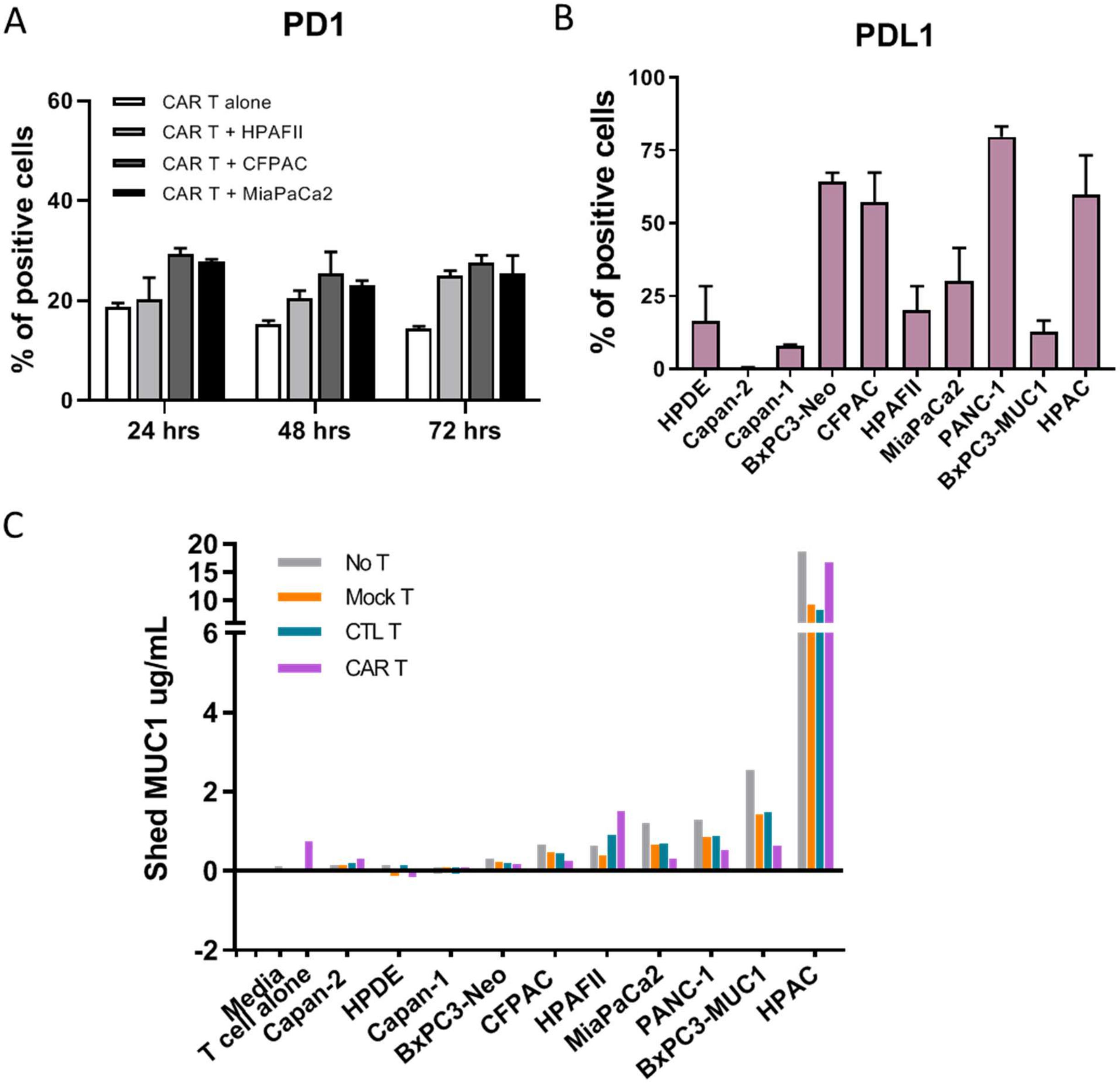

Figure S2. A. PD1 expression on CAR T cells before and after exposure to HPAFII, CFPAC, and MiaPaCa2 cells for 24, 48, and 72 h. Percentage of positive cells was measured using flowcytometry. CAR T cells exposed to PDA cells express higher levels of PD1 compared to unexposed CAR T cells, however there was no significant difference in PD1 expression of CAR T cells when co-cultured with resistant (HPAFII and CFPAC) vs. sensitive cells (MiaPaca2). Error bars, SEM. **B.** PDL1 expression in a panel of PDA cells measured using flowcytometry. There was no correlation between the PDL1 and resistance level in PDA cells. Error bars, SEM. $n = 3$. **C**. Level of shed MUC1 in the 72 h co-culture supernatant of PDA cells and T cells measured by ELISA. High MUC1 expressing cells released higher amount of MUC1 compared to low MUC1 expressing cells. However, this amount is negligible (< 4 ug/mL). HPAC exceptionally released significant amount of MUC1 (~18 ug/mL) into the media before and after exposure to CAR T cells (72 h). Data suggests shed MUC1 does not contribute to the immune resistance of HPAFII and CFPAC. Each bar represents an average of three replicates.

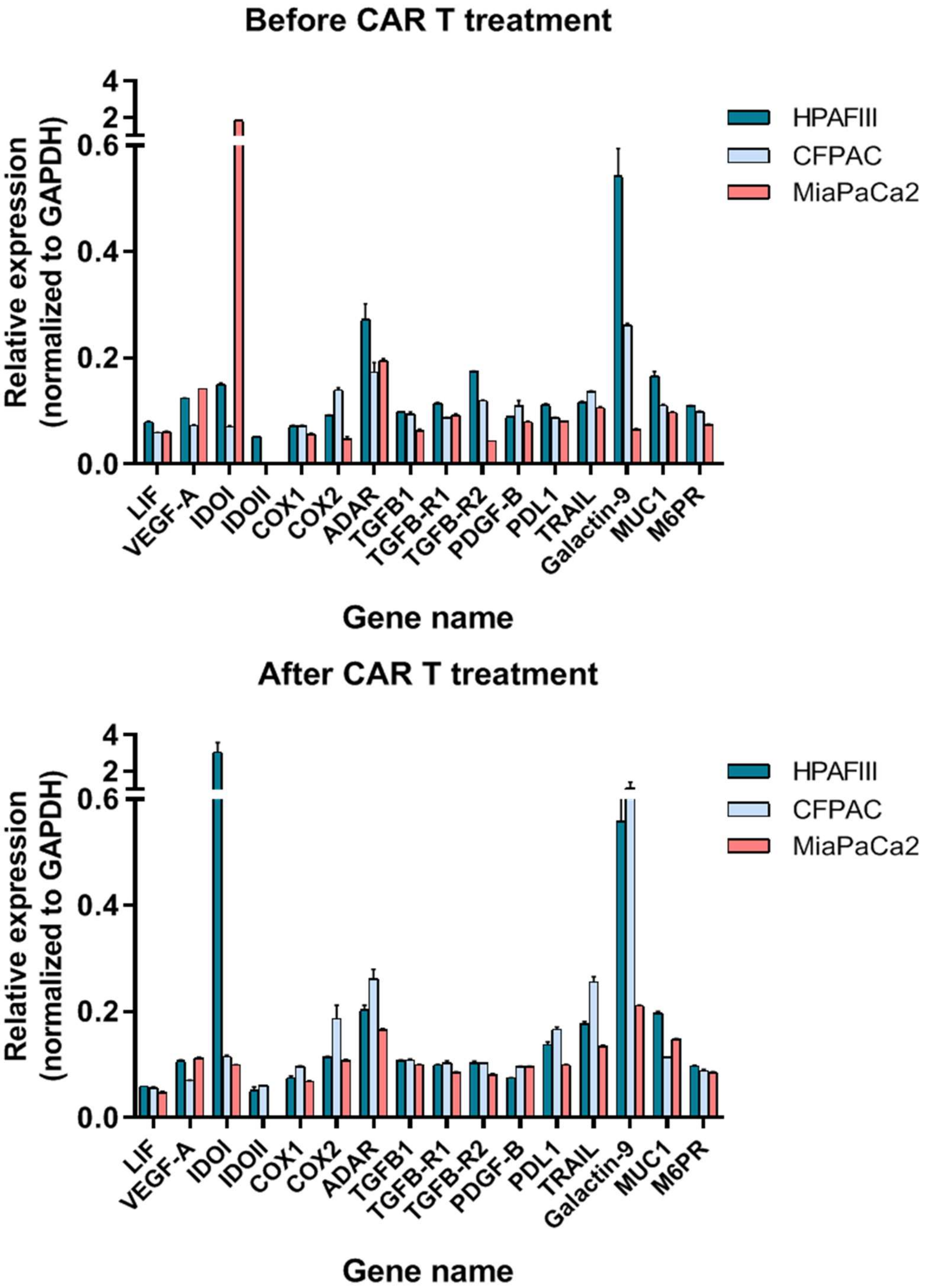

Figure S3. qPCR analysis of 16 resistance related genes in resistant and sensitive PDA cells before and after CAR T cell therapy. Relative expression of the genes compared to GAPDH is shown. Most of the genes were expressed at low level, except IDO1, COX1 and 2, ADAR1, and galectin-9. For more detail, see figure 5C.

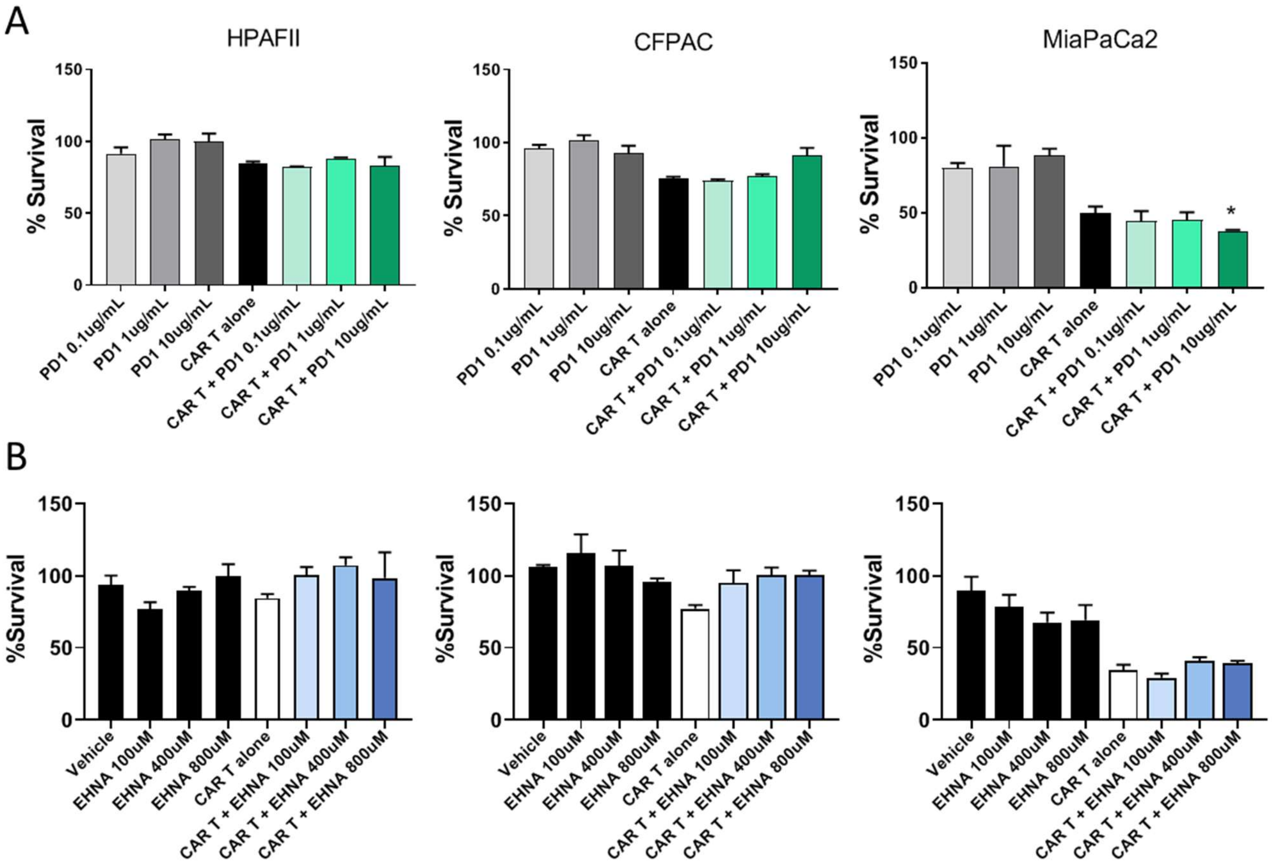

Figure S4. CAR T cell therapy in combination with anti-PD1 blocking antibody and ADAR1 blocking agent (EHNA). **A.** Percentage survival of HPAFII, CFPAC, and MiaPaCa2 PDA cells treated with PD1 blocking Ab alone, T cells alone, and combination, at three different concentrations of Ab. PDA cells were co-cultured with mock or CAR T cells, -/+ anti-PD1 blocking Ab for 72 h at T:E 1:10, and their survival level was measured using MTT assay. Percentage survival was normalized to mock T cells. Resistant cells killing by CAR T cells was not improved by adding anti-PD1 blocking Ab; while MiaPaCa2 cell's killing was improved by the combination therapy. **B.** Percentage survival of HPAFII, CFPAC, and MiaPaCa2 PDA cells treated with EHNA alone, T cells alone, and combination, at three different doses of EHNA is shown. EHNA did not improve cytotoxicity of CAR T cells against resistant PDA cells. Unpaired Student's t-test, comparing CAR T and PD1 or EHNA group to CAR T alone group, * $p = 0.0404$. Error bars, SEM. $n = 4$.

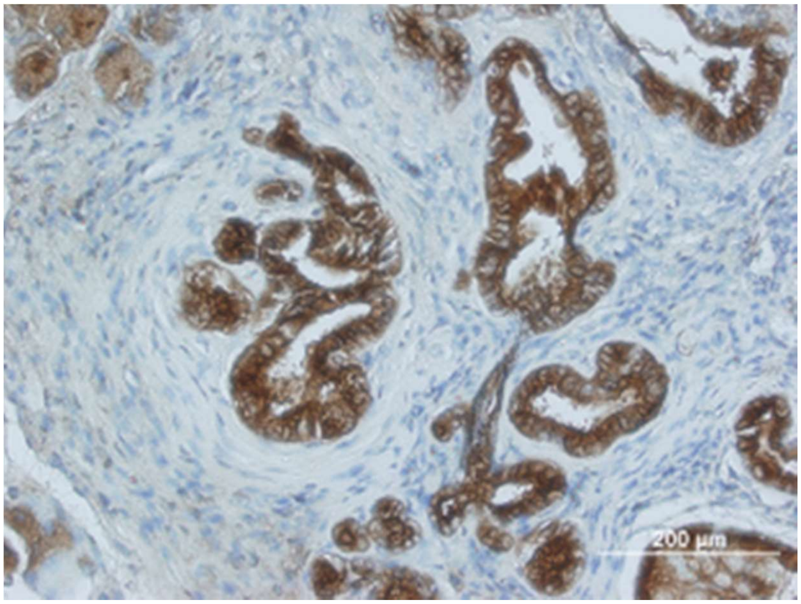

Figure S5A. Selective epithelial detection of tMUC1 using TAB004. PDA tumors from the spontaneous PDA mouse model, PDA.MUC1Tg (also designated KCM), were stained with TAB004 and the stroma shows minimal staining, whereas strong staining was observed in ductal epithelial cells expressing tMUC1, confirming selective epithelial detection of tMUC1.

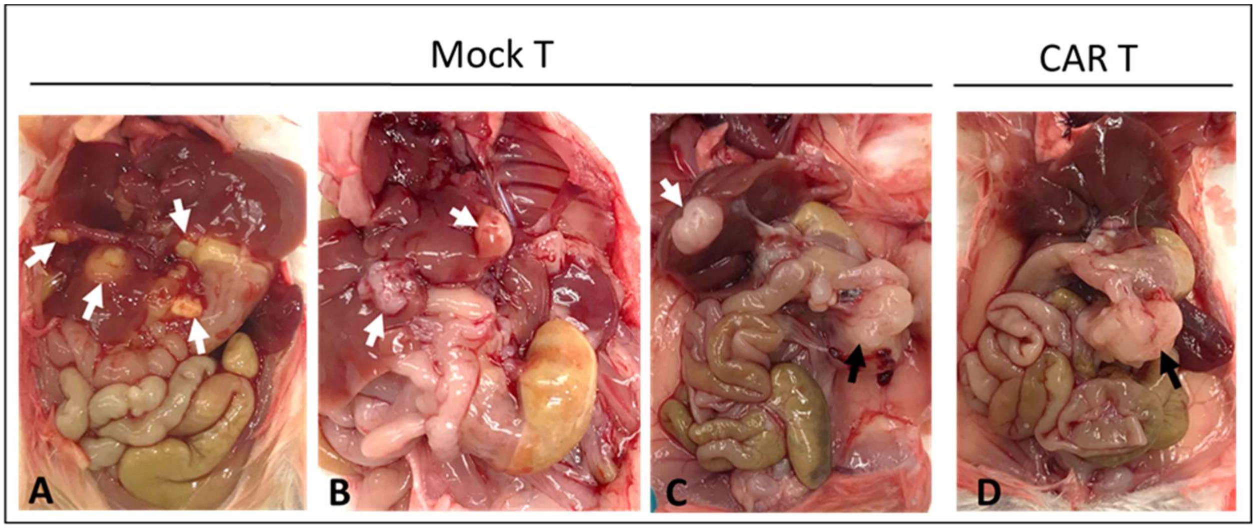

Figure S5. PDA metastasis lesions. Figures taken from mock treated mice at endpoints show metastatic lesions in the abdominal cavity on several organs, mainly liver, stomach, and small intestine. CAR T treated group had no visible metastasis. White arrows indicate metastasis in figure A, B, and C. Black arrows indicate the tumor in C and D. Tumors are covered behind the stomach in A and B.

Cell's name	Origin	Source	Culture condition	Link
AG02102 Normal fibroblast	Endometrium	Biopsy from uterus	Eagle's Minimum Essential Medium with 10%FBS, at 37 ʻC and 5% CO2	https://www.coriell.org/0/Sections/Search/Sample_Detail.aspx?Ref=AG02102&Product=CC
AG02603 Normal fibroblast	Lung	The biopsy was taken post-mortem on 8/4/77. The culture was initiated using explants of minced lung tissue.	Eagle's Minimum Essential Medium with 10%FBS at 37 ʻC and 5% CO2	https://www.coriell.org/0/Sections/Search/Sample_Detail.aspx?Ref=AG02603&Product=CC
AG08789 Normal fibroblast, later named as AG12361	Skin	The biopsy site is the mesial aspect of the mid-upper left arm, taken ante-mortem.	Eagle's Minimum Essential Medium with 10%FBS at 37 ʻC and 5% CO2	https://www.coriell.org/0/Sections/Search/Sample_Detail.aspx?PgId=166&Ref=AG12361
AG11132 Normal breast epithelial	Breast organoid	Biopsy during reduction mammoplasty	MEGM BulletKit (Lonza, Basel, Switzerland)	https://www.coriell.org/0/Sections/Search/Sample_Detail.aspx?Ref=AG11132&Product=CC
AG11134 Normal breast epithelial	Breast organoid	Biopsy during reduction mammoplasty	MEGM BulletKit (Lonza, Basel, Switzerland)	https://www.coriell.org/0/Sections/Search/Sample_Detail.aspx?Ref=AG11134&PgId=166
AG11136 Normal breast epithelial	Breast organoid	Biopsy during subcutaneous mastectomy	MEGM BulletKit (Lonza, Basel, Switzerland)	https://www.coriell.org/0/Sections/Search/Sample_Detail.aspx?Ref=AG11136&PgId=166
AG11137 Normal breast epithelial	Breast organoid	Biopsy during reduction mammoplasty	MEGM BulletKit (Lonza, Basel, Switzerland)	https://www.coriell.org/0/Sections/Search/Sample_Detail.aspx?Ref=AG11137&PgId=166
AG11138 Normal breast epithelial	Breast organoid	Biopsy during mastectomy	MEGM BulletKit (Lonza, Basel, Switzerland)	https://www.coriell.org/0/Sections/Search/Sample_Detail.aspx?Ref=AG11138&PgId=166
BxPC3-Neo Transfected BxPC3 (ATCC® CRL-1687™)	Pancreas	Pancreas	RPMI-1640 supplemented with 10% fetal bovine serum, 1% penicillin/streptomycin and 1% glutamax	https://www.atcc.org/products/all/CRL-1687.aspx
BxPC3-MUC1 Transfected BxPC3	Pancreas	Pancreas	RPMI-1640 supplemented with 10% fetal bovine serum, 1% penicillin/streptomycin and 1% glutamax	https://www.atcc.org/products/all/CRL-1687.aspx

(ATCC® CRL-1687™)				
Capan-1 (ATCC® HTB-79™)	Pancreas	Derived from liver metastatic	RPMI-1640 supplemented with 10% fetal bovine serum, 1% penicillin/streptomycin and 1% glutamax	https://www.atcc.org/products/all/HTB-79.aspx#generalinformation
Capan-2 (ATCC® HTB-80™)	Pancreas	Pancreas	RPMI-1640 supplemented with 10% fetal bovine serum, 1% penicillin/streptomycin and 1% glutamax	https://www.atcc.org/Products/All/HTB-80.aspx#culturemethod
CFPAC (ATCC® CRL-1918™)	Pancreas	Derived from liver metastatic	Dulbecco's modified Eagle's medium (DMEM) supplemented with 10% fetal bovine serum, 1% penicillin/streptomycin and 1% glutamax	https://www.atcc.org/Products/All/CRL-1918.aspx#culturemethod
HPAC (ATCC® CRL-2119™)	Pancreas	Pancreas	DMEM supplemented with 10% fetal bovine serum, 1% penicillin/streptomycin and 1% glutamax	https://www.atcc.org/Products/All/CRL-2119.aspx
HPAFII (ATCC® CRL-1997™)	Pancreas	Pancreas	Minimum Essential Medium (MEM) supplemented with 10% fetal bovine serum, 1% penicillin/streptomycin and 1% glutamax	https://www.atcc.org/Products/All/CRL-1997.aspx
HPDE (H6c7) Normal pancreatic epithelial cells	Pancreas	Normal pancreatic duct cells	DMEM supplemented with 10% fetal bovine serum, 1% penicillin/streptomycin and 1% glutamax	https://www.kerafast.com/product/2101/human-pancreatic-duct-epithelial-cell-line-h6c7
MiaPaCa2 (ATCC® CRL-1420™)	Pancreas	Pancreas	DMEM supplemented with 10% fetal bovine serum, 1% penicillin/streptomycin and 1% glutamax	https://www.atcc.org/products/all/CRL-1420.aspx#generalinformation
PANC-1 (ATCC® CRL-1469™)	Pancreas	Pancreas/duct	DMEM supplemented with 10% fetal bovine serum, 1% penicillin/streptomycin and 1% glutamax	https://www.atcc.org/products/all/CRL-1469.aspx
GP2-293 retroviral packaging cell line (cat# 631458)	Human kidney	HEK 293 derived cells	DMEM supplemented with 10% fetal bovine serum, 1% penicillin/streptomycin and 1% glutamax (ThermoFisher Scientific, Waltham, 70 MA).	Clonetech https://www.takarabio.com/assets/documents/Certificate%20of%20Analysis/631458-101513.pdf

Table S1. Detailed information regarding the cells used in this study.
